# Immunological data from cancer patients treated with Ad5/3-E2F-Δ24-GMCSF suggests utility for tumor immunotherapy

**DOI:** 10.18632/oncotarget.2901

**Published:** 2015-02-24

**Authors:** Otto Hemminki, Suvi Parviainen, Juuso Juhila, Riku Turkki, Nina Linder, Johan Lundin, Matti Kankainen, Ari Ristimäki, Anniina Koski, Ilkka Liikanen, Minna Oksanen, Dirk M. Nettelbeck, Kalevi Kairemo, Kaarina Partanen, Timo Joensuu, Anna Kanerva, Akseli Hemminki

**Affiliations:** ^1^ Cancer Gene Therapy Group, Transplantation Laboratory & Haartman Institute, University of Helsinki, Helsinki, Finland; ^2^ Institute for Molecular Medicine Finland (FIMM), Helsinki, Finland; ^3^ Division of Global Health/IHCAR, Karolinska Institutet, Stockholm, Sweden; ^4^ CSC - IT Center for Science Ltd, Helsinki, Finland; ^5^ Department of Pathology, HUSLAB and Haartman Institute, Helsinki, University Central Hospital and Genome-Scale Biology, Research Programs Unit, University of Helsinki, Helsinki, Finland; ^6^ German Cancer Research Center (DKFZ), Heidelberg, Germany; ^7^ Docrates Cancer Center, Helsinki, Finland; ^8^ Department of Obstetrics and Gynecology, Helsinki University Central Hospital, Helsinki, Finland; ^9^ TILT Biotherapeutics Ltd, Helsinki, Finland

**Keywords:** Oncolytic, immunotherapy, cancer, ATAP

## Abstract

Oncolytic viruses that selectively replicate in tumor cells can be used for treatment of cancer. Accumulating data suggests that virus induced oncolysis can enhance anti-tumor immunity and break immune tolerance. To capitalize on the immunogenic nature of oncolysis, we generated a quadruple modified oncolytic adenovirus expressing granulocyte-macrophage colony-stimulating factor (GMCSF). Ad5/3-E2F-Δ24-GMCSF (CGTG-602) was engineered to contain a tumor specific E2F1 promoter driving an E1 gene deleted at the retinoblastoma protein binding site (“Δ24”). The fiber features a knob from serotype 3 for enhanced gene delivery to tumor cells. The virus was tested preclinically *in vitro* and *in vivo* and then 13 patients with solid tumors refractory to standard therapies were treated. Treatments were well tolerated and frequent tumor- and adenovirus-specific T-cell immune responses were seen. Overall, with regard to tumor marker or radiological responses, signs of antitumor efficacy were seen in 9/12 evaluable patients (75%). The radiological disease control rate with positron emission tomography was 83% while the response rate (including minor responses) was 50%. Tumor biopsies indicated accumulation of immunological cells, especially T-cells, to tumors after treatment. RNA expression analyses of tumors indicated immunological activation and metabolic changes secondary to virus replication.

## INTRODUCTION

Despite progress in cancer prevention, early diagnosis and conventional treatment methods, most metastatic solid tumors remain essentially incurable. One experimental treatment option is oncolytic virotherapy which utilizes the potential many viruses have for replication in tumor cells, followed by lysis. Adenoviruses are the most extensively utilized viruses for gene therapy approaches, and with their well demonstrated safety in thousands of patients, and several positive randomized trials [1–4], they remain a solid platform for innovative therapy approaches. In the field of oncolytic viruses, two randomized trials have been completed, both with positive results. An oncolytic adenovirus improved the efficacy of chemotherapy for treatment of metastatic head and neck cancer [5] and a GMCSF armed oncolytic herpes viruses was effective in the treatment of metastatic melanoma [6]. However, overall survival results have not yet been positive on a statistically significant level and thus room for improvement remains.

Adenoviruses are immunogenic viruses [7], and since it seems that the immune response is a major determinant of the antitumor effect of oncolytic viruses [8, 9], they have a great potential for cancer therapy utilities. Based on the “danger signal” paradigm [10], the presence of oncolytic viruses within a tumor can act as a provocative danger signal for the immune system. Further, tumor associated antigens (TAAs) are released by oncolysis for presentation to the immune system in an environment conducive for immunity [11–13]. Arming adenovirus with immunostimulatory molecules has been utilized as an approach to further augment immune responses against tumor antigens.

The design of the novel oncolytic virus Ad5/3-E2F-Δ24-GMCSF incorporates three concepts.

I) Serotype 3 knob for enhanced entry into tumor cells. Most clinical trials performed with adenoviruses thus far are based on serotype 5 or the closely related serotype 2 [4]. The primary receptor for these viruses is the coxsackie adenovirus receptor (CAR) [14] which is often down-regulated in aggressive human tumors [15]. Enhanced delivery to and killing of cancer cells, clinical specimens and xenograft tumors in mice is achieved when the native Ad5 fiber knob is replaced by that of serotype 3 virus, without loss of safety in humans [16, 17]. Supporting the safety of Ad3 components, a completely serotype 3 based oncolytic adenoviruses have shown potential in preclinical [18] and clinical settings [19].

II) E2F1 promoter and Δ24 deletion of viral E1A for efficient and specific replication in tumor cells. Although previous constructs such as CGTG-101 [9], CGTG-102 [16] and CGTG-103 [20], featuring a single “delta-24” mutation for tumor selectivity, have been safe in patients, there may be advantage for multiple levels of control. Tumor selectivity with “delta-24” occurs after E1A expression, and thus E1A is expressed even in normal cells. E1A can cause toxicity and it may contribute to anti-viral immunity, and there may be some “leaky” activation of late viral proteins, again possibly resulting in toxicity or anti-viral immunity. Thus, it would be appealing to control expression of the mutated E1A with a tumor specific promoter [21]. The human E2F1 promoter is active in most tumor cell lines mutated in the pRb pathway [22, 23] thus facilitating the use of the E2F1 promoter in most if not all tumors [24, 25].

III) Granulocyte macrophage colony-stimulating factor (GMCSF) is a widely used immunostimulatory molecule in oncolytic viruses in clinical settings, now also proven effective in a randomized, global phase 3 melanoma trial which met its primary endpoint of durable response rate [6, 26]. It is a potent inducer of systemic anti-tumor immunity associated with recruitment and maturation of antigen presenting cells (APCs), mainly dendritic cells, as well as recruitment of cells of the innate immunity arm, including natural killer cells and neutrophils. In immunocompetent Syrian hamsters virally produced GMCSF has been able to activate anti-tumor immune responses, enhancing the efficacy of oncolytic adenovirus [9, 27], and human data is compatible with these notion [9, 16, 20, 28]. An advantage of local production of cytokines is that high concentrations can be achieved where useful (at the tumor site) while retaining lower systemic levels, which can cause adverse events or untoward immunological consequences such as recruitment of myeloid derived suppressor cells [16, 29].

## RESULTS

### Preclinical experiments

**Figure 1:**
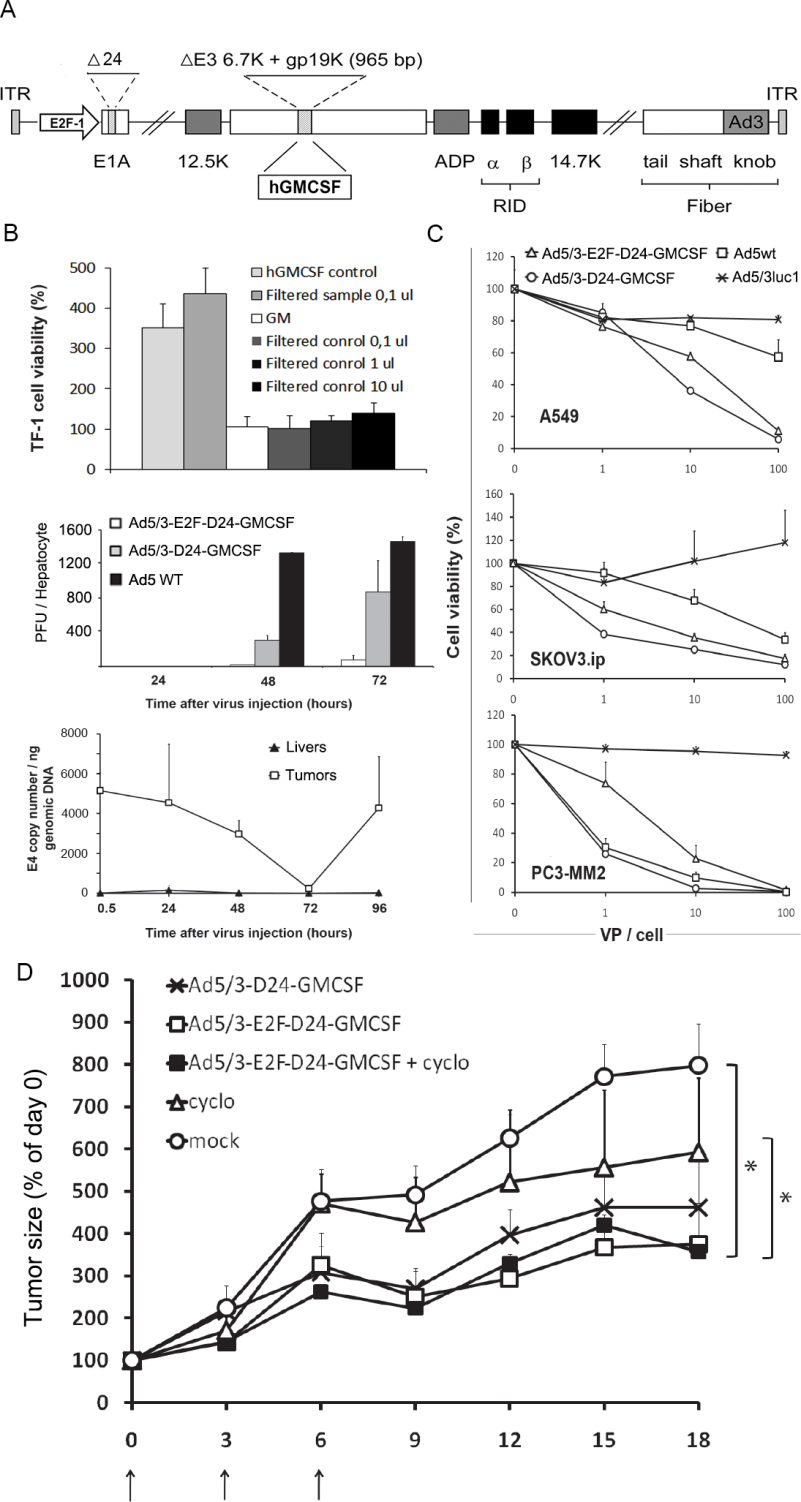
(A) A schematic representation of the Ad5/3-E2F-Δ24-GMCSF virus The *E1A* promoter is replaced by a human *E2F1* promoter, which controls the transcription of the *E1A* gene. The *E1A* gene features a 24 base-pair deletion to avoid the self-activation of the promoter by E2F released by E1A-Rb interaction, a critical fault in previous designs with an intact E1A gene. Gp19k and 6.7K in *E3* have been replaced with the cDNA of human GM-CSF. The serotype 5 (Ad5) fiber knob has been replaced by the serotype 3 (Ad3) knob. **(B)** Top panel: Ad5/3-E2F-Δ24-GMCSF -expressed GMCSF is functionally active. TF1 cells, which require human GMCSF for viability, were cultured in the presence of human recombinant GMCSF or filtered supernatant from virus infected cells. As a control, supernatant from cells infected with a virus not expressing GM-CSF was used (Ad5/3-Δ24). The viability of TF1-cells treated with growth medium (GM) only was set as 100% hGMCSF control and filtered supernatant showed significantly (*P* < 0.001) higher viability over GM, while no difference was seen between other groups. Middle panel: Less infective virus particles were produced by Ad5/3-E2F-Δ24-GMCSF in human primary hepatocytes at 48 and 72 h time points compared to the control viruses, indicating low replication in normal human cells. Bottom panel: to evaluate tumor selectivity of the virus, livers of non-tumor-bearing Syrian hamsters were injected and no viral E4 copy number increase was detected with qPCR. In contrast, E4 copy number increased in HapT-1 tumors. Replacing the native E1A promoter with the E2F-1 promoter does not impair virus replication and cell killing effect *in vitro* **(C)** or *in vivo* **(D)**. (c) Results of a cell viability assay shows the cell killing efficiency of Ad5/3-E2F-Δ24-GMCSF compared to Ad5/3-Δ24-GMCSF - a control virus bearing the genetically intact E1A promoter - a wild type serotype 5 virus and a non-replicating control virus Ad5/3luc1 in A549 lung cancer cells, SKOV3ip.1 ovarian cancer cells and PC3-MM2 prostate cancer cells. (d) Ad5/3-E2F-Δ24-GMCSF significantly (*P* < 0.01) slowed down tumor progression compared to the mock (growth medium only) animals in Syrian Hamsters bearing pancreatic cancer tumors. 3 × 10^8^ VP of virus was administered intratumorally on days 0, 3 and 6. Concomitant low dose cyclophosphamide did not significantly improve the anti-tumor effect of Ad5/3-E2F-Δ24-GMCSF.

The E1A region, E3 region and fiber are genetically modified in Ad5/3-E2F-Δ24-GMCSF (Figure 1A). Virally produced GMCSF was tested functional by analyzing the growth of GMCSF dependent TF-1 erythroleukemia cells upon addition of filtered supernatant from Ad5/3-E2F-Δ24-GMCSF infected A549 cells (Figure 1B top).

The selectivity of Ad5/3-E2F-Δ24-GMCSF was studied in primary human hepatocytes. The levels of infective viral particles remained 15 to 11 fold lower than with the parental oncolytic control virus without the E2F1 promoter (replication controlled only by the 24bp deletion) and 65 to 19 times lower than in wild type Ad5 infected hepatocytes at 48 and 72 hours, respectively (Figure 1B middle).

To study the selectivity of viral replication in Syrian hamsters, a model reported semipermissive for human adenovirus [16], tumors and livers were collected following virus injection and analyzed for virus copy number (Figure 1B bottom). A secondary peak seen in virus copy number in tumors suggests effective virus DNA replication, while particles remained low in the liver at all time points, suggesting lack of replication in normal cells.

The *in vitro* efficacy of Ad5/3-E2F-Δ24-GMCSF was evaluated on three cancer cell lines representing non-small cell lung, ovarian and prostate cancer (Figure 1C). Ad5/3-E2F-Δ24-GMCSF achieved cancer cell killing efficacy comparable to the isogenic control without the E2F1 promoter and wild type Ad5, while being superior to the non-replicative Ad5/3luc1 (*P* < 0.05), which implies retained oncolytic potency despite quadruple genetic modification.

*In vivo* potency and selectivity of Ad5/3-E2F-Δ24-GMCSF was characterized in an immunocompetent Syrian hamster model previously reported semi -permissive for adenoviral replication [30, 31]. Subcutaneous hamster pancreatic cancer (HapT1) tumors were treated with intratumoral injections (Figure 1D) and Ad5/3-E2F-Δ24-GMCSF restricted tumor progression significantly when compared to cyclophosphamide only or to mock (growth medium only) treated groups. The recent discovery that not all hamster tumor cell lines are fully permissive to 5/3 chimeric virus – in comparison to human substrates – may have impacted efficacy in this experiment [32]. To evaluate the ability of Ad5/3-E2F-Δ24-GMCSF to cause oncolysis of HapT1 cells, a cell killing assay was performed. Interestingly, and in contrast to many other hamster cell lines [28], HapT1 did not allow for productive oncolysis (Figure S1) and therefore the anti-tumor effect was caused by immune response towards tumor cells filled with virus components (virus DNA replicated) and surrounded by GMCSF.

### Patients

Thirteen patients with advanced metastatic tumors refractory to and progressing after standard therapy were treated with 2–4 rounds of Ad5/3-E2F-Δ24-GMCSF (Table 1). Treatments were performed in a personalized manner and 1–10 tumor sites were injected. Patient C332 received only 1 round of treatment and then underwent pre-planned surgery. Most patients had received multiple lines of chemotherapy before virus treatments and were progressing, thus constituting a highly treatment refractory patient population (Table S1). There was variation between tumors and patients, and thus the patient population well represents “real-life” patients with advanced cancer [33–35].

**Table 1:** Patients at baseline

Patient ID	Age (y)	Sex	Diagnosis	WHO	Virus Dose**	Treatment rounds
O314	62	F	Ovarian cancer	1	1–3 × 10^11^	3
O337	69	F	Ovarian cancer	2	5 × 10^11^	3
O340	74	F	Ovarian cancer	0	5 × 10^11^	3
O351	72	F	Ovarian cancer	2	3 × 10^11^	3
C312	54	M	Rectum cancer	1	1–3 × 10^11^	3
C332*	49	F	Colon cancer	0	8 × 10^11^	1
H192	54	M	Pancreatic cancer	1	3 × 10^11^	2
H344	58	F	Pancreatic cancer	1	8 × 10^11^	3
I347	51	M	Melanoma	2	5 × 10^11^	3
R319	67	F	Breast cancer	1	3–5 × 10^11^	3
R342	54	F	Breast cancer	2	3 × 10^11^	2
R356	40	F	Breast cancer	1	1 × 10^12^	4
S352	59	F	Sarcoma	1	3–10 × 10^11^	3
S354	50	F	Fibrosarcoma	2	3 × 10^11^	4

* Hyperthermic intraperitoneal chemotherapy (HIPEC) surgery 3 weeks later, thus included only for immunohistochemistry analysis.

** First treatment was given at the lower indicated dose. All patients (excluding C332) received low dose cyclophosphamide to reduce T-reg cells, patients H192, H344, I347, S352 and S354 received also low dose pulse temozolomide to enhance autophagy resulting from oncolysis.

### Safety of the treatments

Table S2 summarizes the adverse reactions recorded during all 39 treatment rounds. Grade 1–2 flu like symptoms, fever, fatigue and pain were experienced in more than half of the treatments. Most grade 3 events were self-limiting or treatable as outpatient, and no grade 4 or 5 adverse effects were observed.

### Virus replication

All patients evaluated for the presence of Ad5/3-E2F-Δ24-GMCSF in serum were negative prior to therapy (*N* = 11, Table 2). One day after the first treatment 8/13 patients had measurable virus genomes, with the highest titer being 1141 VP/ml. 2/4 patients sampled on days 3–8 were positive, with the highest titer of 11523 VP/ml serum, and both (O340 and H344) showed an increase in titer after day 1, suggestive of virus replication.

**Table 2:** Responses, survival and amounts of virus and neutralizing antibodies

	Virus load in serum (copy number) or Neutralizing Antibody Titer (NAb)											Treatment responses		
Patient ID	Days post treatment											PET response (%)	Marker	Survival (days)
		after 1st			after 2nd			after 3rd						
		0	1	3–8	0	1	3–8	0	1	3–10	14–55			
O314	Virus	0	0		0	< 500			0	0	0		mPR	71
	NAb	1						1024						
O337	Virus	0	< 500			0		0	< 500				mSD	276
O340	Virus	0	1141	3608	0	< 500	0	0	< 500	0	0	MMR (–10%)	mCR	890
	Nab	4096					4096							
O351	Virus		0		0	< 500	0	0	0	0			mMR	87
	NAb	256							4096					
C312	Virus	0	< 500		0	< 500			< 500			SMD (+23%)	mPD	397
	Nab	4096						4096						
H192	Virus	0	0		0	< 500							mPD	80
	Nab	256				4096								
H344	Virus	0	< 500	11523	0	< 500	0	0	< 500	0	0	PMD	mPD	133
	Nab	256					4096							
I347	Virus	0	< 500	0	0	< 500	0	0	< 500					106
	Nab	64			4096									
R319	Virus	0	0	0	0	0		0	< 500			PMR (–49%)	mPR	332
R342	Virus	0	< 500			< 500							mPD	76
R356	Virus	0	< 500		< 500	876			< 500				mPR	102
	NAb	1							1024					
S352	Virus	0	0		0	< 500	0	0	0		0	SMD (+6%)		112
	NAb		16					1024						
S354	Virus	0	789		0	0			< 500		0	CMR (–76%)^a^		1009*
	NAb	256							4096		4096			

MMR = minor metabolic response, SMD = stable metabolic disease, PMD = progressive metabolic disease, PMR = partial metabolic response, CMR = complete metabolic response.

a complete metabolic response, 76% reduction in tumor size

* patient alive on 14.1.2014 mPR = marker partial response, mSD = marker stable disease, mCR = marker complete response, mMR=marker minor response, mPD=marker progressive disease.

Typically, titers less than 500 VP/ml were seen 1 day after the second and third treatments while no virus (*N* = 21) was seen on other days after the second and third treatments. These data are compatible with replication of the virus in tumors, especially subsequent to the first injection, with less release of genomes into the circulation occurring after 2nd and 3rd injection.

### Neutralizing antibodies and anti-hexon antibodies

At baseline, 4 patients had a low titer (1–64) of anti-Ad5/3 neutralizing antibodies (Table 2). 4 patients had an intermediate titer (256) while 2 patients had a high titer (4096) already at baseline. Thus, at baseline the median titer was 256 and by the time of the second treatment the titer had risen (*P* < 0.01) to a median of 4096 and stayed there until the end of follow-up.

To provide an alternative view on anti-viral antibodies, we also analyzed patient serums for anti-hexon IgG, hexon being the main capsid protein of the virus. At baseline, all patient serums had low titers (between 30 and 200U/ml) of anti-hexon IgG. Three weeks after the first treatment all patients showed increase in serum antibody titers (titers between 200 and 3000). Titers stayed elevated with a slow decreasing trend in some patients, during the months following treatment (Figure S2A). Further, ascites samples from patient O314 were analyzed for anti-hexon antibody titers at baseline and 3 weeks after treatment and antibody levels increased from 57 U/ml to 509 U/ml (Figure S2B). Titers of antibodies attached to ascites cells were also evaluated, and they increased from 42 to 471 U/100 mg protein. The latter may indicate virus replication, and subsequent antibody binding to cells present in ascites, a typical location of ovarian cancer cells.

### PET CT and tumor markers

All patients had progressing tumors prior to treatment. 6 patients could be assessed with PET-CT (modified PERCIST criteria [36]) (Table 2). Response was typically assessed 3–4 weeks after the last virus injection; typically 3 injections were given 3 weeks apart. R319 had partial metabolic response (PMR, 49% reduction in metabolic activity) in the injected liver tumor and a complete metabolic response in a non-injected mediastinal tumor (Figure 4a), S354 had a complete metabolic response (CMR, Figure 4b and Figure S3), O340 had a minor metabolic response (MMR), C312 and S352 had stable metabolic disease (SMD) and H344 had progressive metabolic disease (PMD). Therefore, the radiological disease control rate (stable disease or better) was 83% while the PET response rate (including MMR) was 50%.

With regard to tumor markers, assessed for patients who had elevated markers at baseline, 3/10 patients had reduction of marker levels (O314, O337, O351), 2/10 had initial reduction and subsequent elevation of marker levels (R356, R319), 1 patient had initial elevation and subsequent reduction (O340) and 4/10 had elevation of marker levels (H344, C312, R342, H192, Figure S4). Thus, overall, 6/10 patients had some indication of possible treatment benefits, as measured by tumor markers in serum. R319, O340, H344 and C312 were evaluated also with PET-CT. While R319 and O340 had partial and minor metabolic responses, C312 had only stabilization (and in fact a 23% increase in SUVmax, fulfilling criteria of SMD) and H344 had metabolic progression. Thus, there was rather good correlation between PET response and tumor marker data.

Overall, with regard to tumor marker or radiological responses, signs of antitumor efficacy were seen in 9/12 evaluable patients (75%). These patients lived a median of 135 days while the median survival of the other three was 80 days. In patient I347 treatment efficacy could not be evaluated with any method. Thus, in an overall intent-to-treat analysis, there was some evidence of efficacy in 9/13 patients (69%). Overall survival of all patients is shown in (Figure S5).

### Changes in peripheral blood T-cell activity are seen in patients treated with Ad5/3-E2F-Δ24-GMCSF

**Figure 2:**
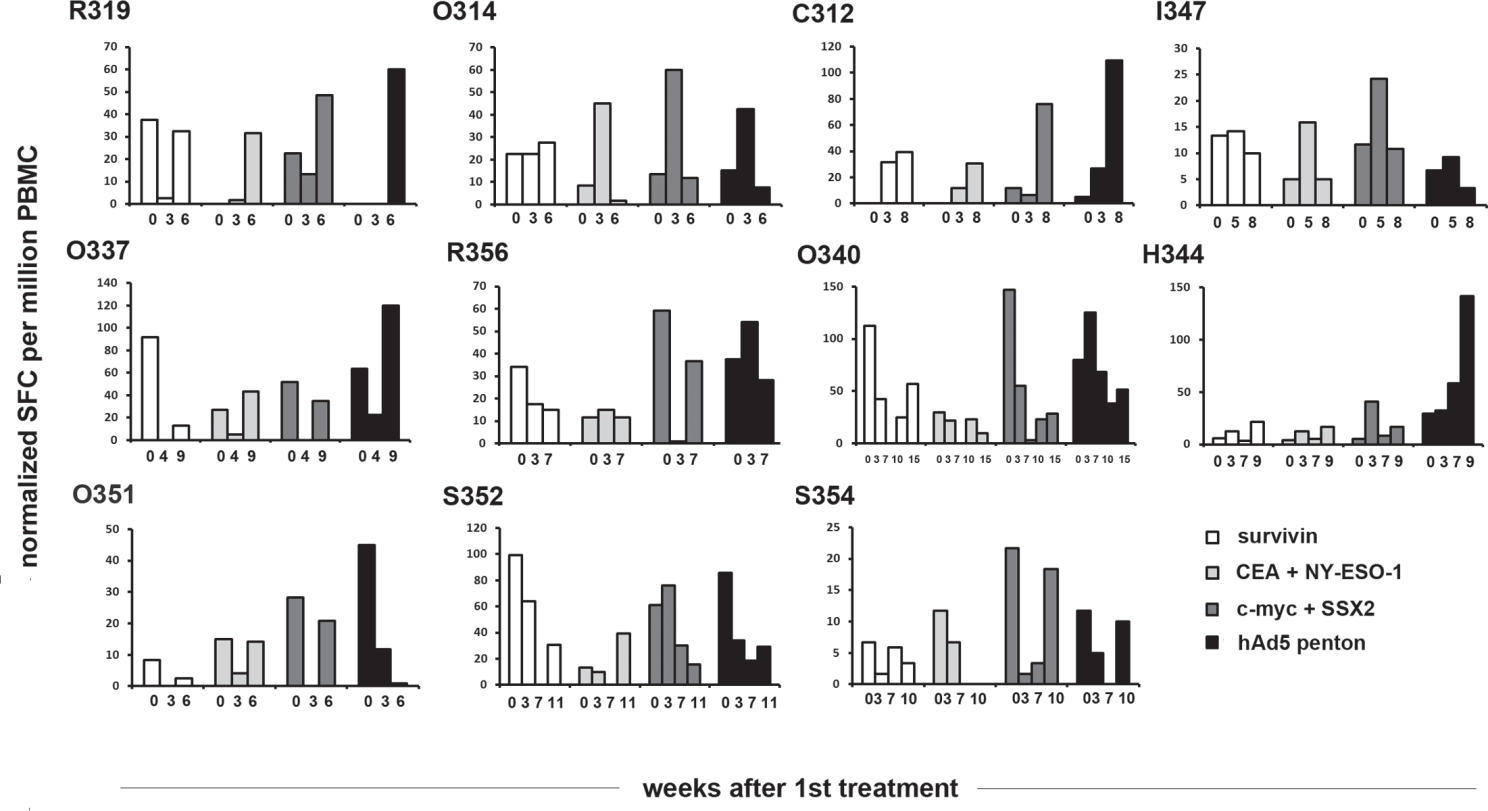
Ad5/3-E2F-Δ24-GMCSF elicited T cell responses against adenovirus and tumor epitopes in cancer patients PBMCs harvested from patients treated with Ad5/3-E2F-Δ24-GMCSF were analyzed by IFN-gamma ELISPOT upon stimulation with a mix of peptide from Adenovirus 5 and mixes of peptides from tumor antigens CEA and NY-ESO-1 (pool1), c-myc and SSX2 (pool2) and Survivin alone. Bars represent the frequency of IFN-gamma producing peptide specific PBMCs per million cells. SFC, Spot Forming Colonies.

Peripheral blood mononuclear cells (PBMCs) were collected and pulsed with Ad5 penton peptide or with tumor associated peptide pools (Survivin, CEA + NY-ESO-1 or c-myc + SSX2). IFN-γ production was then analyzed with ELISPOT to evaluate the number of anti-tumor and anti-viral T-cells (Figure 2). Interestingly, in most patients the behavior of anti-tumor cells closely resembled changes seen in anti-viral cells; if the latter increased, also the former did and vice versa. In 8 patients (R319, O314, C312, I347, O337, R356, H344, S352), there was an increase (considered as a higher than at baseline at any timepoint) in one or more classes of anti-tumor T-cells, while in 3 patients (O340, O351, S354) there was a decrease. For anti-viral T-cells, the respective numbers were also 8 and 3 (increase: R319, O314, C312, I347, O337, R356, O340, H344, decrease: O351, S352, S354) and thus there was concordance in 9/11 (82%) patients between anti-tumor T-cells and anti-viral T-cells (the discordant patients were O340 and S352). This finding may suggest that in humans, anti-viral response corresponds and may contribute to anti-tumor response through epitope spreading and reduction of tumor-associated immunological tolerance. [17] However, as proposed before [17, 37], there was no correlation between changes in T-cells and clinical indicators of treatment efficacy, as a decrease of T-cells in blood might indicate trafficking to tumors [17, 38]. T-cell anergy [38] (no induction, no trafficking) was not seen in any patients.

### Antibodies against tumor associated antigens decrease in responding patients

Antibodies against tumor associated antigens are frequently elevated in cancer patients even at early stages of the disease, indicating recognition of tumor epitopes by the immune system [39]. Interestingly, a body of data from the cancer vaccine field indicates that anti-tumor antibodies can be indicative of treatment efficacy. Specifically, a decrease in anti-tumor antibodies can indicate anti-tumor response and an increase can indicate lack of response [40]. For example, antibodies against NY-ESO-1 were shown to disappear from serum of patients that are in remission [41].

**Figure 3:**
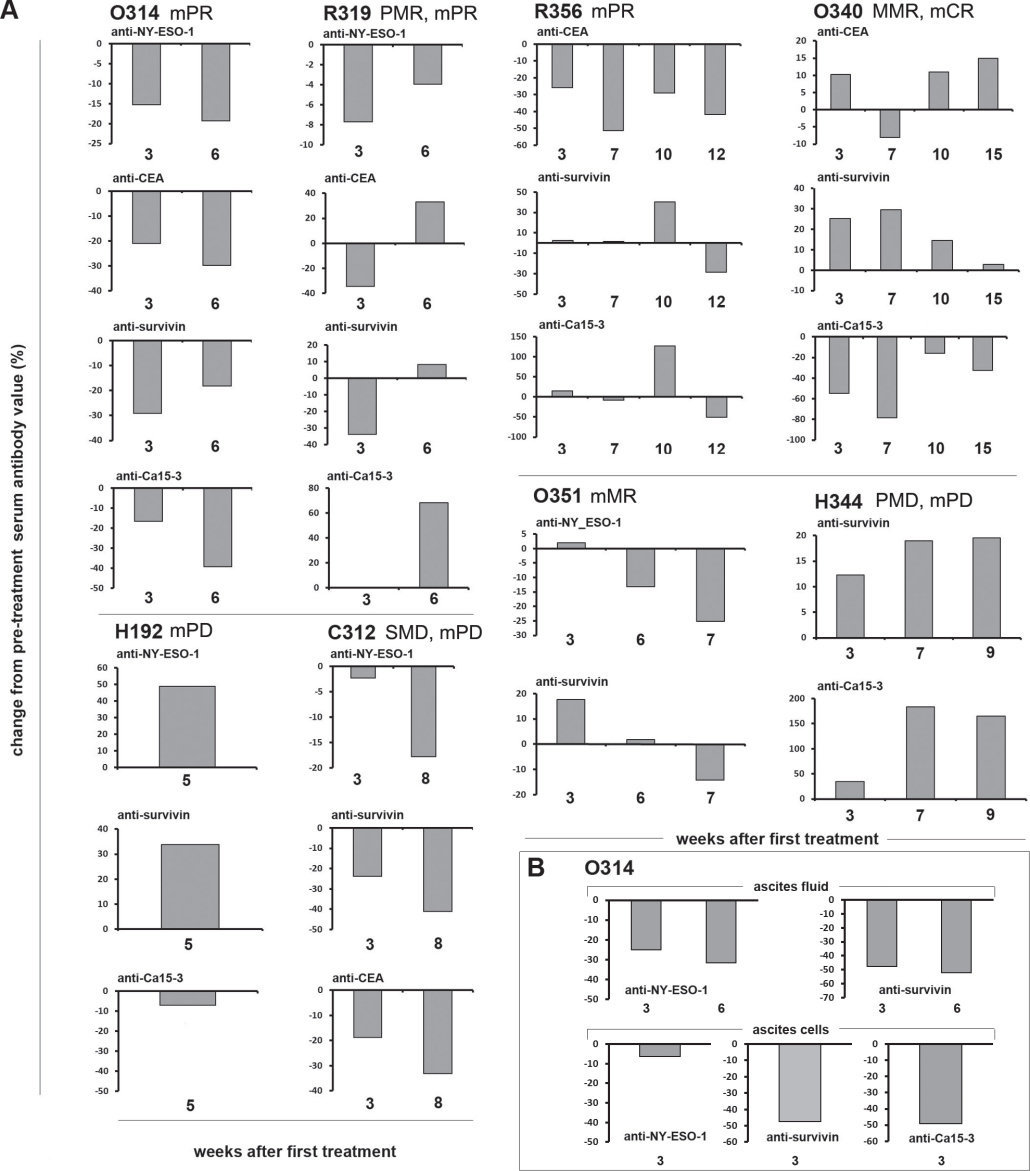
**(A)** Antibodies against tumor associated antigens (TAAs) NY-ESO-1, CA-15–3, CEA and survivin were analyzed from patient serum (a) and ascites fluid and cells of patient O314 **(B)** before and after viral treatment, and the data is presented as proportional change (%) of antibody levels (as estimated by ELISA absorbance units) from pre-treatment values **(A)** Treatment often resulted in decrease of elevated levels of antibodies against TAAs in patients that showed a concomitant response in PET-CT or decrease in marker levels (patients O314, R356, R319, O340, C312, O351). Patients H192 and H344 did not show any benefit in PET-CT or markers and increasing levels of antibodies against TAAs were noted. **(B)** Malignant ascites (resulting from peritoneal tumor masses) was removed from the peritoneal cavity of patient O314 before and 19 and 40 days after virus administration. As with the serum samples decreasing amounts of antibodies against TAA were noted.

In our patients, antibodies against CEA, NY-ESO-1, survivin or MUC-1 frequently decreased in patients with signs of anti-tumor efficacy (Figure 3A). For example, patient O314 seemed to benefit from treatment as she had a partial response in CA12–5. She had a decrease in all four anti-tumor antibodies measured in her blood. R319 had a partial metabolic response in PET and a partial tumor marker response, and decrease in 3/4 anti-tumor antibodies in her blood. R356 had a partial marker response and a decrease in all three anti-tumor antibodies. Likewise, there seemed to be correlation between clinical benefits and anti-tumor antibodies in C312 and O351. In contrast, in patients H192 and H344, there were no signs of clinical benefits, and in both cases anti-tumor antibodies increased. Overall, a significant (*P* = 0.02) correlation between anti-tumor antibodies and clinical signs of benefit from treatment was seen.

**Figure 4:**
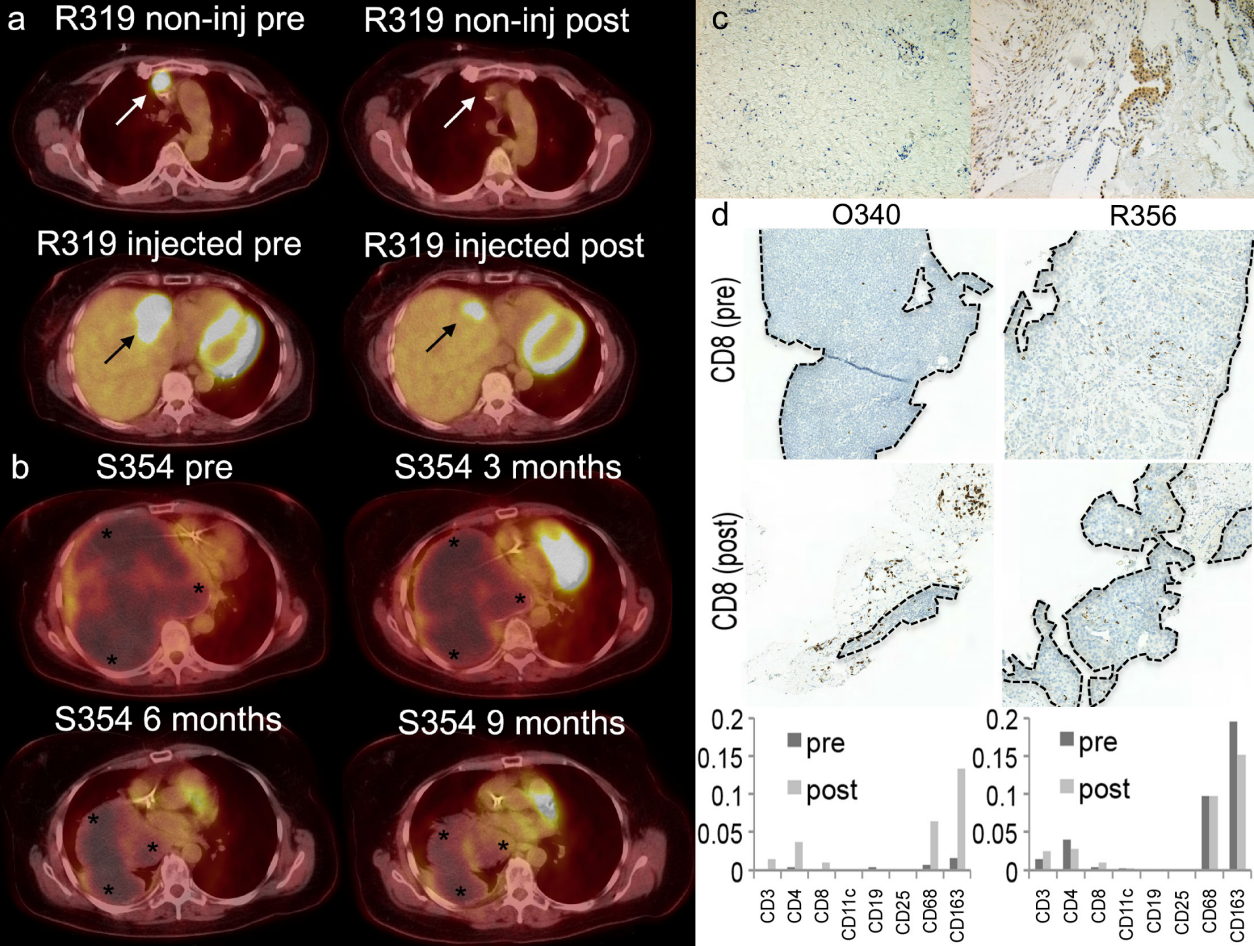
Positron emission tomography-computed tomography (PET-CT) fusion images from patients R319 **(a)** and S354 **(b)** **(a)** A non-injected mediastinal lesion disappeared after treatment (white arrows). 49% reduction in metabolic activity of an injected lesion in the liver (black arrow). **(b)** A total of 76% reduction is seen in tumor (stars) volume of patient S354. A 46% reduction in tumor volume was seen at 3 months, 76% reduction at 6 months and a stable situation at 9 months after the first treatment. Patient S354 was given 3 treatments every 3 weeks and after that a continuation treatment scheme was started with treatments given every 3 weeks. **(c)** Patient C322 was operated 3 weeks after treatment with Ad5/3-E2F-Δ24-GMCSF. More CD8+ cytotoxic T-cells (brown) were seen in tumor (right panel) than normal tissue (left panel) collected during the operation. **(d)** Biopsies before the first virus treatment and three weeks after the third virus treatment were available from patients O340 and R356. Immunohistochemical CD8 staining (dark brown dots) is shown. The area of the tumor was drawn by a pathologist and marked by a dotted line in the figure. Amount of stained cells in the tumor area divided by the area containing tumor is shown in the graph below. Large amounts of immunological cells seem to arrive to the tumor after the treatment. Also large quantities are seen around the tumor rim. Especially profound is the T-cell infiltration within the tumor of patient O340 that had a lower quantity of immunological cells at baseline.

In one case (O314), anti-tumor antibody levels in blood could be compared to anti-tumor antibody levels in ascites and cells present in ascites (which typically contains tumor cells). Interestingly, there was close correlation between all three compartments, suggesting that systemic measurement of antibody levels could be a reliable indicator of local events at the tumor (Figure 3B), which is compatible with the long half-life and systemic dissemination of antibodies in general.

### Biopsies

Patient C322 underwent surgery 3 weeks after treatment with Ad5/3-E2F-Δ24-GMCSF, which allowed immunohistochemical analysis of the operated tumor. More CD8+ cytotoxic T-cells (brown) were seen in tumor than in normal tissue (Figure 4c).

Biopsies before the first virus treatment and three weeks after the third virus treatment were available from patients O340 and R356 (Figure 4d). Before treatment both of the core needle biopsies taken from patient O340 and R356 showed only tumor tissue. In contrast, after treatment biopsies showed only a proportion of tumor tissue (O340 circa 15%, R356 circa 75% tumor cells). Assuming similar skills by the radiologist on both occasions, one explanation for this finding would be treatment mediated anti-tumor effects.

Immunohistochemical stainings for various immunological cell types were performed (Figure 4d). Interestingly with patient O340 a clear increase in the proportion of mature T-cells (CD3) (x34), T-helper cells (CD4) (x9) and cytotoxic T-cells (CD8) (x26) is seen, while CD11c-positive cells, B-cells (CD19) and CD25-positive cells (including eg. regulatory T-cells) virtually disappeared from the tumor. Concurrently the number of monocytic macrophages (CD68 and CD163) increased (x10 and x8 respectively). This patient seemed to respond to treatment as measured by PET-CT (MMR), markers (mCR), long survival (890d) (Table 2) and decrease in anti-Ca15–3 antibodies (Figure 3). Moreover, a drastic decrease in tumor antigen specific T-cells was observed in blood preceding the biopsy (Figure 2). As proposed [17], a decrease in anti-tumor T-cells in blood is compatible with trafficking of those cells to the tumor, a notion supported by evidence in this case, as CD8+ cells increased 26-fold in the tumor.

Less dramatic changes in cell density were also seen in the immunohistochemical stainings of patient R356 (up to x3). A partial response (mPR) in markers was observed but the patient survived only 102 days (Table 2). Some changes were seen in blood T-cells and anti-tumor antibodies (Figure 2–3). Taken together, the data suggest that in this patient the anti-tumor effect was transient, perhaps chiefly mediated by oncolysis, since a clear immunological change did not occur at the tumor, as estimated by immunohistochemistry of the biopsy.

In these two cases with biopsy material available, we also analysed RNA expression patterns and predicted underlying biological functions by using Ingenuity pathway analysis software (Ingenuity System Inc, USA) (Figure S6). Thousands of pathways were analyzed, but only 25 (O340) and 18 (R356) were found significantly altered by treatment. Interestingly, nine of these pathways were the same in both patients, possibly hinting at mechanism of action. Noteworthy were the three retinoid X receptor (RXR) pathways, with known associations to immunology, particularly CD8+ and regulatory T-cell responses [42, 43]. These and “acute phase response signaling” pathways seem to suggest that treatment with Ad5/3-E2F-Δ24-GMCSF induces immunological activity.

## DISCUSSION

Based on the preclinical and clinical findings reported here, Ad5/3-E2F-Δ24-GMCSF seems to function as designed: The Ad3 fiber knob enables efficient transduction of tumor cells while the E2F1 promoter and 24bp deletion provide excellent tumor selectivity (as demonstrated by lack of replication in hamster livers *in vivo* and human hepatocytes *ex vivo*). Functional GMCSF is produced following cellular entry.

Interestingly, as reported before with this HapT1 Syrian hamster tumor model with a similar although distinct virus [16] it seems that after the virus has entered the tumor it takes 3–4 days for the viral DNA copy number to peak after which a much lower amount of virus DNA is present in the tumor, probably because infected cells were killed either due to oncolysis or immunological eradication of infected cells. Seemingly in support of the former, the amount of viral DNA again starts to increase after dipping, which is compatible with a second wave of viral replication (Figure 1B bottom). However, lack of productive oncolysis *in vitro* with this cell line (Figure S1) suggests in fact that *in vivo* killing of cells filled with virus genomes and other components is in fact immunological. Our conclusions are that a) Ad5/3 is not oncolytic on Syrian hamster HapT1 cells *in vitro* (although the virus is oncolytic in some other Syrian hamster cell lines [28]), b) DNA replication is nevertheless seen *in vivo*, c) *in vivo* efficacy is caused mostly by immune response against the virus and/or stimulated by GMCSF. It seems unlikely that the virus would be oncolytic *in vivo* but not *in vitro* and thus we believe the qPCR result merely indicates DNA replication, not productive oncolysis, thus pin-pointing the block in permissivity between genome replication and lysis of the cell. The waves of DNA replication seen by qPCR must therefore reflect GMCSF stimulated immunological clearance of cells with a high content of adenoviral components.

While the design of this virus might facilitate intravenous administration we have not found a good animal model to test this. Syrian hamsters are technically challenging (fur, thick skin, lacking tail veins) with regard to an intravenous route and mouse tissues are non-permissive for human adenovirus [44]. Variance in cell killing with serotype 5 wild type compared to our fiber modified 5/3 virus in different cell lines (Figure 1C) might be due to the relative expression of the main Ad5 receptor coxsackie and adenovirus receptor versus the presence of Ad3 receptors such as desmoglein 2 as published [45–47].

Treatments were safe and many patients with variable types of solid cancers refractory to other therapies seemed to show signs of efficacy when evaluated by PET-CT or by tumor markers. PET-CT was chosen as the imaging method as we believe the immune reaction caused by treatment results in tumor swelling which restricts the utility of size based evaluation criteria [36].

No virus DNA was detected in blood after the 2nd and 3rd injections, suggesting tight restriction of oncolysis to the tumors, or less replication than after the 1st cycle, or both. Interestingly, as reported with preclinical data [28] we noted disappearance of a non-injected tumor with patient R319 (Figure 4a). These findings are in support of the immunogenic mechanism being more important than pure oncolysis with regard to efficacy.

The patents had quite advanced tumors and they were heavily pretreated often with multiple lines of chemotherapy. Thus, when oncolytic virus treatment was stopped, some patients progressed rapidly, while some stabilized for extended times. It should be noted that most patients progressed off therapy, and thus eventual progression is not really evidence of lack of efficacy. However, as this was not a randomized trial we would hesitate to make any assumptions on efficacy over control patients. There are many well-known caveats in comparisons to historical controls and thus we believe a randomized setting is the appropriate approach for assessing effects on overall survival.

Interestingly, PET-CT and tumor marker responses and survival (Table 2) seemed to correlate with: 1) changes in the numbers and activity of circulating tumor reactive lymphocytes (Figure 2); 2) a decrease in anti-tumor antibodies in blood and ascites (Figure 3); 3) increases in CD3, CD4, CD8, CD68 and CD163 positive cells at the tumor (Figure 4).

Although these preliminary findings need to be confirmed in larger patient cohorts, immunohistochemical analysis of tumor biopsies suggested that immunological cells accumulated at tumors in patients responding to treatment while at the same time changes in cancer specific T cells in blood could be seen. This is compatible with previously published data and suggestive of trafficking of anti-tumor (and anti-viral) cells from blood into tumors [17, 46, 48–51]. Viral DNA was often detected in serum for several days after the first treatment while after subsequent treatments qPCR tended to be negative. Antibody titer against the virus increased exponentially while antibodies against tumor associated antigens decreased in responding patients, a finding proposed associated with anti-tumor efficacy [40, 41].

At baseline patient R356 had more (CD3 x33, CD4 x10, CD8 x9, CD11c x21, CD68 x15, CD163 x12) immunological cells in the tumor than patient O340 (Figure 4). After treatment the number of immunological cells increased more with O340 than with R356. Since both tumors were progressing at baseline, the data might indicate that the tumor of R356 featured stronger local immunosuppression (regulatory T cells etc.) to counterbalance the more prominent immunological infiltrate. For such patients virus induced immunological response might not be as useful, as the “gas pedal” is already fully engaged. For such tumors, “releasing the brake” might be more useful instead, alone or in combination with oncolytic immunotherapy. Thus new drugs such as PD-1 or anti-CTLA-4 antibodies combined to oncolytic viroterapy and/or T-cell therapy might lead to additive efficacy. It is clear that more patients are needed to study these phenomena in more detail and no definite conclusions can be made from a small number of biopsies, but on the other hand the data reported here may be the first available information on potentially important aspects of the technology.

In summary, Ad5/3-E2F-Δ24-GMCSF (CGTG-602) seems safe and effective for treatment of cancer. Clinical trials would be useful for demonstrating these aspects in more homogeneous patient populations, and for collecting more biological samples, to extend upon the preliminary analyses done here.

## MATERIALS AND METHODS

### Viruses and cancer cell lines

The construction of Ad5/3-E2F-Δ24-GMCSF is described in supplementary materials and methods. Ad5/3-Δ24, Ad5/3lucI and Ad5/3-D24-GM-CSF have been described [16, 46, 52]. Cell lines used herein and their testing have been described [18, 53].

### Preclinical experiments

*In vitro assays.* GMCSF dependent TF-1 erythroleukemia cells (Sigma Aldrich, St Louis, MO) were used to evaluate the functionality of GMCSF as described [16]. The results constitute of an average of 7 (mock 21, hGMCSF 11) samples, students T-test was used for statistics. The experiment showed that virus-produced hGMCSF was as bioactive as the commercial positive control (Recombinant human GM-CSF, 2ng/ml, PeproTech, 300–03) as reported [9, 28, 53–56]. Oncolytic potency was tested with MTS-assay performed 6 days (A549 and PC3-MM2) or 14 days (SKOV3.ip1) after virus incubation when 100% cell killing with the highest viral dose was observed [18]. Shown results are the average of four samples, error bars represent standard deviation. Students T-test was used for statistical analyses. Selectivity of virus replication in human primary hepatocytes is described in supplementary materials and methods. *In vivo* efficacy. Hamster pancreatic cancer tumors (HapT1) were grown subcutaneously in Syrian hamsters as described [9]. At 7 days tumors were injected with 3 × 10^8^ VP/tumor of either of Ad5/3-E2F-Δ24-GMCSF (*n* = 6) or Ad5/3-Δ24-GMCSF (*n* = 5) 3 times every 3 days. Further, 5 animals received cyclophosphamide intraperitoneally (2 mg/hamster) in combination with Ad5/3-E2F-Δ24-GMCSF, 5 hamsters received cyclophosphamide only and 4 animals were mock-treated with intratumoral growth medium injections. Tumor volume was calculated by using a formula of (0.5 × length) × (width^2^).

*In vivo* tumor selectivity of Ad5/3-E2F-Δ24-GMCSF replication. After hamster HapT1 tumors reached approximately 0,5 cm in diameter (7 days), 3 × 10^8^ VP of Ad5/3-E2F-Δ24-GMCSF was injected intratumorally (*n* = 8 tumors/timepoint). Hamsters without tumors (*n* = 2 hamsters/timepoint) were injected directly into the liver. Animals were killed and tumors or livers collected 0.5, 24, 48, 72 or 96 hours after virus injection, snap-frozen and stored at –80°C. Quantitative PCR has been described [16]. Activity of human GM-CSF in Syrian hamsters has been described previously [9, 28, 53–56].

### Patient treatments

Patients were treated in an Advanced Therapy Access Program, regulated by EC/1394/2007 on advanced therapy medicinal products, amending Directive 2001/83/EC and Regulation (EC) No 726/2004. According to EC/1394/2007 manufacturing of advanced therapy medicinal products shall be authorized by the competent authority of the Member State, which in Finland is the Finnish Medical Agency (FIMEA). FIMEA also requires reporting of adverse reactions. Virus administration was performed by ultrasound-guided intratumoral injection and circa one fifth of the dose was given intravenously during the first treatment only. The rationale for the latter relates to the generally low to average amount of neutralizing antibodies that are typically present at baseline, while after treatment antibodies often increase rapidly and thus the value of subsequent intravenous dosing is unknown. In any case, there is no need for subsequent intravenous injection because the tumor produces virus which is then shed into blood. Thus in subsequent injections the whole dose was given intratumorally. Most patients were treated three times every three weeks. Doses of 1–10×10^11^ VP were used based on safety results previously published with Ad5/3-Δ24-GMCSF, a related virus lacking the E2F1 promoter [57]. All patients exluding C332 received low dose cyclophosphamid for reduction of regulatory T-cells [58]. Patients H192, 344, I347, S352 and S354 received also low dose temozolomide for enhancement of autophagy during oncolysis [59]. Typically, cyclophosphamide was used throughout the treatments with a dose of 50mg/day while temozolomide was used 5–7 days before and/or 7–14 days after the treatment at 100mg/day. Neither approach is known to have anti-tumor activity on their own [37, 60]. Written informed consent was obtained from all patients. Patients were monitored overnight at the clinic and then as outpatients for 28 days for adverse events. Adverse reactions were recorded according to Common Terminology for Adverse Events v3.0. Survival and late adverse events were followed ad infinitum. ATAP treatments are not a trial but an individualized treatment program. However, in a separate research project, collection and analysis of biopsies was approved by the HUCH operative ethics committee, and a separate informed consent procedure was employed to ensure separation of research from treatment (Dnro 368/13/03/02/2009). Other patient sample analyses are also approved by the local Ethics Committee (HUS 62/13/03/02/2013). Because many cancer patients have symptoms due to disease, preexisting symptoms were not scored if they did not become worse. However, if the symptom became more severe, e.g., pretreatment grade 1 changed to grade 2 after treatment, it was scored as grade 2. The neutralizing antibody assay [61] and the determination of virus genomes in blood [16] are previously published. Interferon gamma ELISPOT is described [17], all results were compared to cells spontaneously producing the cytokine, i.e. PBMCs without stimulation. For detail on the immunohistocehmical stainings, tumor biopsies and their quantitation please refer to supplementary materials and methods.

## SUPPLEMENTARY MATERIALS AND METHODS


