# Supplementary material for: Immunological data from cancer patients treated with Ad5/3-E2F-Δ24-GMCSF suggests utility for tumor immunotherapy
Source: Oncotarget. 2015 Feb 24;6(6):4467–81. doi: 10.18632/oncotarget.2901 (PMC4414204; doi:10.18632/oncotarget.2901)
Supplement: Supplementary file 1 [file oncotarget-06-4467-s001.pdf]

SUPPLEMENTARY MATERIALS AND METHODS

Viruses

For construction of Ad5/3 E2F Δ24 GMCSF, the E2F 1 promoter was amplified by PCR with specific primers with restriction enzyme cutting sites for NotI and XhoI designed so that the promoter could be inserted into pSE1.D24 [62] to control *E1A*. The resulting plasmid, pE2F.E1.D24, contains the E2F-1 promoter controlling *E1A* gene that has a 24 bp deletion in constant region 2. pAd5/3-E2F.D24-GMCSF was generated by homologous recombination in *E.coli* between PmeI-linearized pE2F. E1.D24 and *Srf*I-linearized rescue plasmid pAd5/3-D24-GMCSF[16]. The Ad5/3E2FΔ24GMCSF virus genome was released by *Pac*I digestion and transfection to A549 cells for amplification and rescue. All phases of the cloning were confirmed by multiple PCRs and restriction digestions as well as sequencing for the relevant areas of the plasmids. All phases of the virus production, including transfection, were done on A549 cells to avoid risk of wild type recombination. Viruses were purified on cesium chloride gradient centrifugation.

In vitro selectivity of Ad5/3E2FΔ24GMCSF in human primary hepatocytes

A 24-well plate was coated with 0.5 mg/ml rat tail type 1 collagen in 1mM acetic acid for 30 minutes. 1.5 x 10^5 primary human hepatocytes (Lonza Ltd, Basel, Switzerland) were seeded per well in the recommended medium with 2% serum. Cells were allowed to rest for 3 hours in 37°C and the growth media was changed to medium without serum. 24 hours later the hepatocytes were infected with 10 VP/cell of viruses. Growth media was changed after 2 hours of infection. Cells and supernatant were collected 24, 48 and 72 hours after infection and frozen at –80°C. Cells and supernatant went through 4 cycles of freezing and thawing prior to the subsequent plaque assay, performed with serially diluted (10^{-1} to 10^{-11}) supernatant from the samples centrifuged for 25 minutes at 4000 rpm prior to infection. Each analysis contained a mock infected well for comparison. Two independent experiments (each with 7 replicates) were performed and the average and standard deviation is shown in the figure. Students *T*-test was used for statistics.

Anti-TAA assay

For the Anti-TAA assay ELISA was used as the detection method. Actual antibody levels are not obtained, but the results are represented as means of optical density. As a conjugant, anti IgG conjugated with alkaline phosphatase was used, and the differences in a colour reaction are interpreted as differences in the amount of serum antibodies bound to the specific protein in each reaction (wells are coated with CEA, NY-ESO-1, surviving or Ca15-3). Higher optical density represents more bound antibodies. Samples were analyzed in triplicates.

Immunohistochemistry

Tumor samples from patient C332 were obtained during a preplanned operation 3 weeks after treatment with Ad5/3E2FΔ24GMCSF. Tumor and normal peritoneal lining were fixed in 4% formalin and paraffin blocks were made. For analysis of CD8 positive cells, i.e. cytotoxic T-cells, tissue sections of 4 µm thickness were prepared, deparaffinized, rehydrated and incubated with a primary mouse anti-human CD8 antibody (NCL-CD8-4B11; Novocastra, Newcastle Upon Tyne, United Kingdom) at a dilution of 1:25 in antibody diluent S0809 (DakoCytomation, Carpinteria, CA, USA). Sections were washed and incubated with a secondary anti-mouse antibody labeled with horseradish peroxide (HRP) and counterstained for hematoxyline.

Biopsies of patients O340 and R356 were collected with biopsy gun. Tissue blocks were sectioned using conventional histological techniques. Serial sections (3,5 µm) were taken and mounted on electrically charged glass slides (SuperFrost Plus, Menzel-Gläser, Germany). The first set of sections was stained with hematoxylin and eosin (H&E) and further sets were used for immunohistochemistry. The immunohistochemical stainings were performed with Ventana Benchmark XT (Tucson, Arizona, USA). Instructions of the manufacturer were followed and the following antigens were used: CD3 (Ventana, 790-4341), CD4 (Cell marque, 104R-16), CD8 (Novocastra, NCL-CD8-4B11), CD11c (Novocastra, NCL-L-CD11c-563), CD19 (Serotec, MCA2454), CD25 (Novocastra, NCL-CD25-305), CD68 (Dako, M0876), CD163 (Novocastra, NCL-CD163). Detection was performed with Ultraview (Ventana, Roche 760-500), incubation time 16min/36°C, DAB- kromogen time 8min/36°C.

Quantification of immunohistochemical stainings

A color information based image processing methodology was applied to quantify the immunohisto-chemical stainings. The samples were first digitally scanned, annotated for tumorous regions and then automatically analyzed.

The slides were digitally scanned with an automated whole-slide scanner (3DHISTECH Ltd, Pannoramic 250 FLASH, Budapest, Hungary) using a Plan-Apochromat 20× objective (numerical aperture 0.8) and a VCC-F52U25CL camera (CIS, Tokyo, Japan) equipped with a 1,624×1,224 pixel 3 Charge Coupled Device (3CCD) sensor. The pixel size of the 3CCD chip is 4.4 µm × 4.4 µm, and combined

with the 20× objective the image resolution is 0.22 µm/pixel. Images were then compressed to a wavelet file format (Enhanced Compressed Wavelet, ECW, ER Mapper, Erdas Inc, Atlanta, Georgia) and archived online using WebMicroscope whole-slide image management platform (WebMicroscope, Fimmic Ltd, Helsinki, Finland) running with image server software (Erdas Apollo Image Web Server, Intergraph, Norcross, GA).

An experienced pathologist inspected the samples and identified tumor regions. A graphics-editing program (Adobe Photoshop CS6, Adobe Systems, Mountain View, California, U.S.) was used to mark up the pathologist's annotations on a 1:8 downscaled image version of the original digital samples. However, the full resolution sample was used in histological inspection and decision making.

For the image processing pipeline, the digitized sample images were downscaled to 1:4 of the original resolution, corresponding now to pixel size of 0.88 µm. Uneven background illumination was corrected by applying a morphological top-hat filtering to the lightness channel (Hue, Saturation and Lightness color model). Based on the standard color deconvolution [63], a monochrome channel (C_{DAB}) was extracted, identifying the image pixels stained with DAB and their staining intensities. A threshold value was defined for the C_{DAB} monochrome image to further detect exclusively positively stained cellular regions and to filter out possible unspecific staining.

Finally, the immunohistochemical samples were quantified by calculating a fraction of positively stained cellular region in the whole region-of-interest, i.e. tumor region. The image-processing pipeline was implemented in matrix laboratory (MATLAB, version R2012b) numerical computing environment.

RNA extraction and microarrays

Biopsies of patients O340 and R356 were stored in RNALater (Life Technologies) until RNA extraction. Total RNA was extracted from the samples using TRIZOL Reagent (Life Technologies) and sequentially purified with the RNAEAsy Mini kit (Qiagen) according standard procedures. Finally, RNA was eluated to 30 µl of RNase-free water (Nalgene). RNA quantity was evaluated spectrophotometrically by using Nanodrop (ThermoFisher Scientific), and the quality was assessed with the Agilent 2100 Bioanalyzer (Agilent Technologies). Normally, samples with no evidence for RNA degradation (RNA integrity number >8) were kept for further experiments.

Genome-wide gene expression profiling of RNA samples was done by hybridizing the RNA to the Illumina HumanHT-12 v4 Expression BeadChips arrays. The labeling and hybridization was performed with Illumina TotalPrep RNA Labeling Kit according to manufacturer´s instructions (revision D (Ambion) Whole-Genome Gene Expression Direct Hybridization Assay Guide, Rev A). BeadChips were washed, blocked and stained with streptavidin-Cy3 and scanned with Illumina iScan by using manufacturer provided protocols. Genome Studio software was used to control the quality of the data.

Microarray data interpretation and pathway analyses

Raw probe level intensity values were summarized and exported with Illumina probe annotations using Illumina BeadStudio. The non-normalized, non-background corrected data were then quantile-normalized and log2 transformed using functions from the beadarray package [64] Finally, probes were annotated according to information retrieved from EnsEMBL using functions from the biomart package [65]. Probes mapped to same EnsEMBL gene were averaged into single expression estimate. Probes pointing to multiple genes or left without a gene information were removed.

For pathway analyses normalized expression values were used to describe gene expression differences before and after Ad5/3E2FΔ24GMCSF treatment. The "Core Analysis" function included in Ingenuity pathway analysis (Ingenuity System Inc, USA) was used to interpret the microarray data in the context of biological processes, pathways and networks. Additional filtering (minimum 1-fold change at log2-value) was applied to eliminate genes with small changes in expression levels. Those genes with known gene reference number (Ensembl) and their corresponding expression log-2 were uploaded into the software. Each gene symbol was mapped to its corresponding gene object in the Ingenuity Pathways Knowledge Base.

After the analysis, generated results were ranked based on significance. Canonical pathways analysis identified the pathways, from the Ingenuity pathway analysis library of canonical pathways, which were most significant to the input data set. The significance of the association between the data set and the canonical pathway was determined based on two parameters: (1) A ratio of the number of genes from the data set that map to the pathway divided by the total number of genes that map to the canonical pathway and (2) a *P* value calculated using Fischer's exact test determining the probability that the association between the genes in the data set and the canonical pathway is due to chance alone.

SUPPLEMENTARY FIGURES AND TABLES

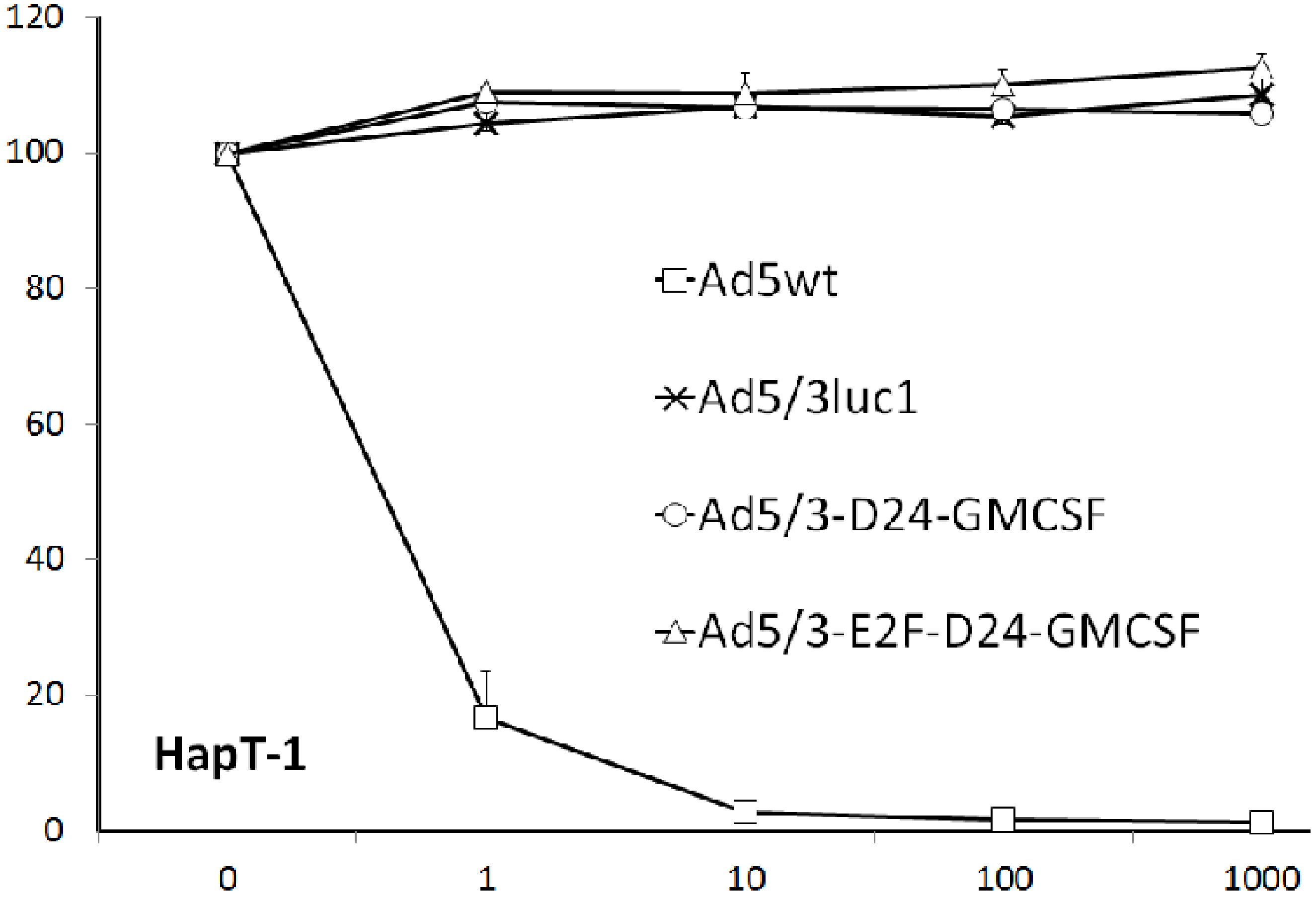

Supplementary Figure S1: MTS assay of HapT-1 cell line (hamster pancreatic cancer) from day 11. Productive *in vitro* oncolysis was not seen with Ad5/3 oncoytic viruses with this hamster cell line. Another assay with similar results was followed to day 21 (data not shown). Average of triplicates is shown. Bars indicate standard deviation.

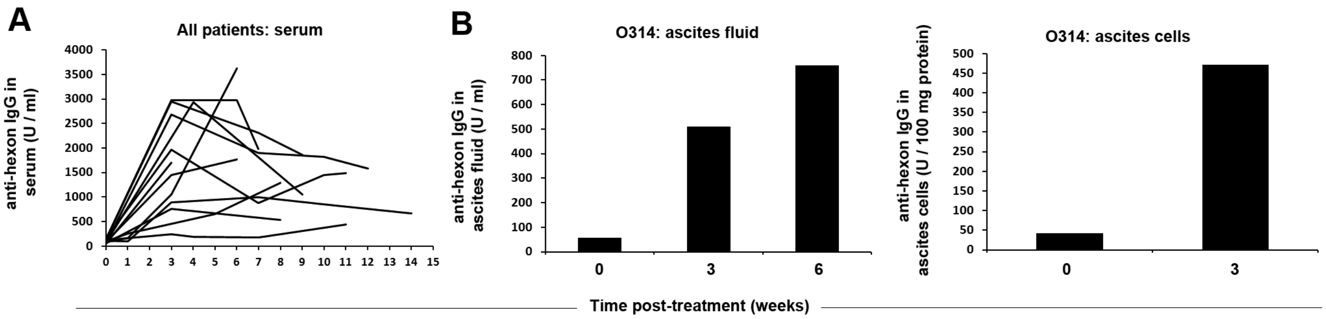

Supplementary Figure S2: Antibody levels against serotype 5 hexon were analyzed from the serum of all patients (A) and ascites fluid and cells of patient O314 (B) before and after viral treatment. (A) In all patients, serum levels were low before treatment but were dramatically elevated after treatment. **(B)** Ascites cells from patient O314 showed a value of 43 U/100 mg before and 471 U/100 mg after treatment, suggesting presence of Ad5/3-E2F-Δ24-GMCSF virus in the malignant cells. Ascites fluid values were elevated from 58 U/ml before treatment to 758 U/ml after treatment.

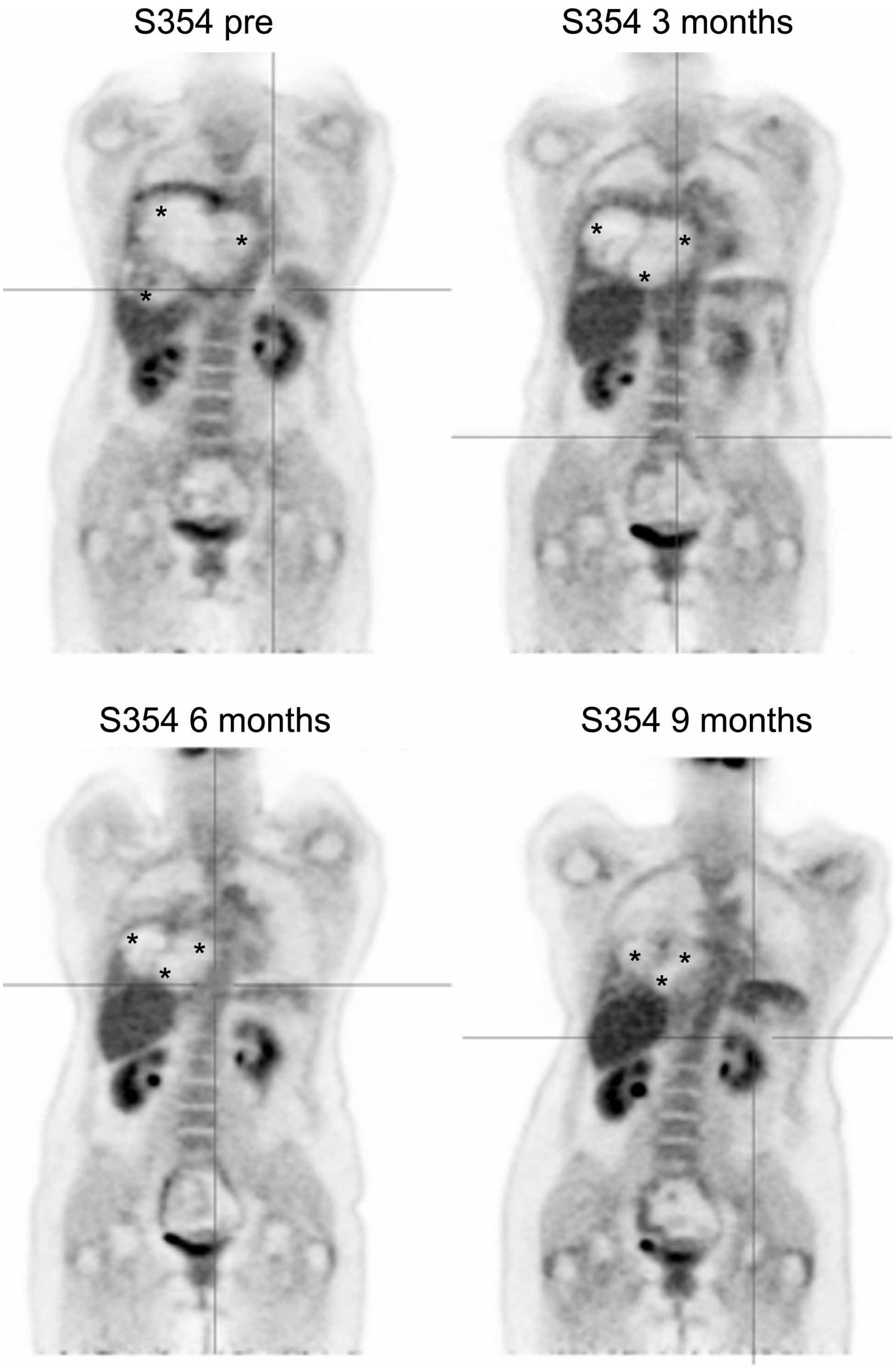

Supplementary Figure S3: Axial CT scans of patient S354. Note anatomical deformities of the mediastinum and heart due to tumor (stars) expansion over the thoracic midline at baseline for patient S354.

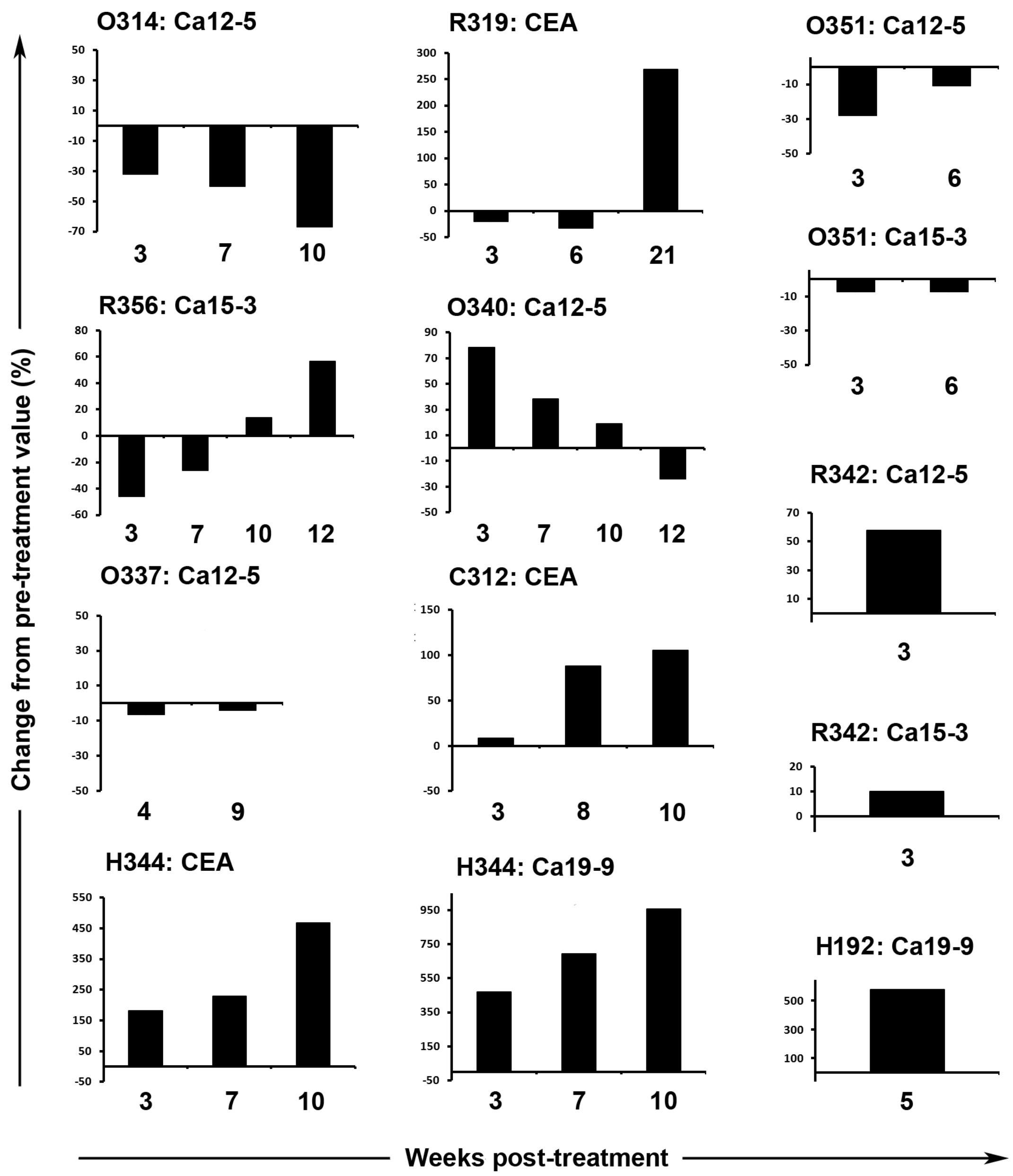

Supplementary Figure S4: Serum marker levels for CEA, Ca12-5, Ca15-3 and/or Ca19-9 were analyzed before and after viral treatment, and the data is presented as proportional change (%) of marker from pre-treatment value.

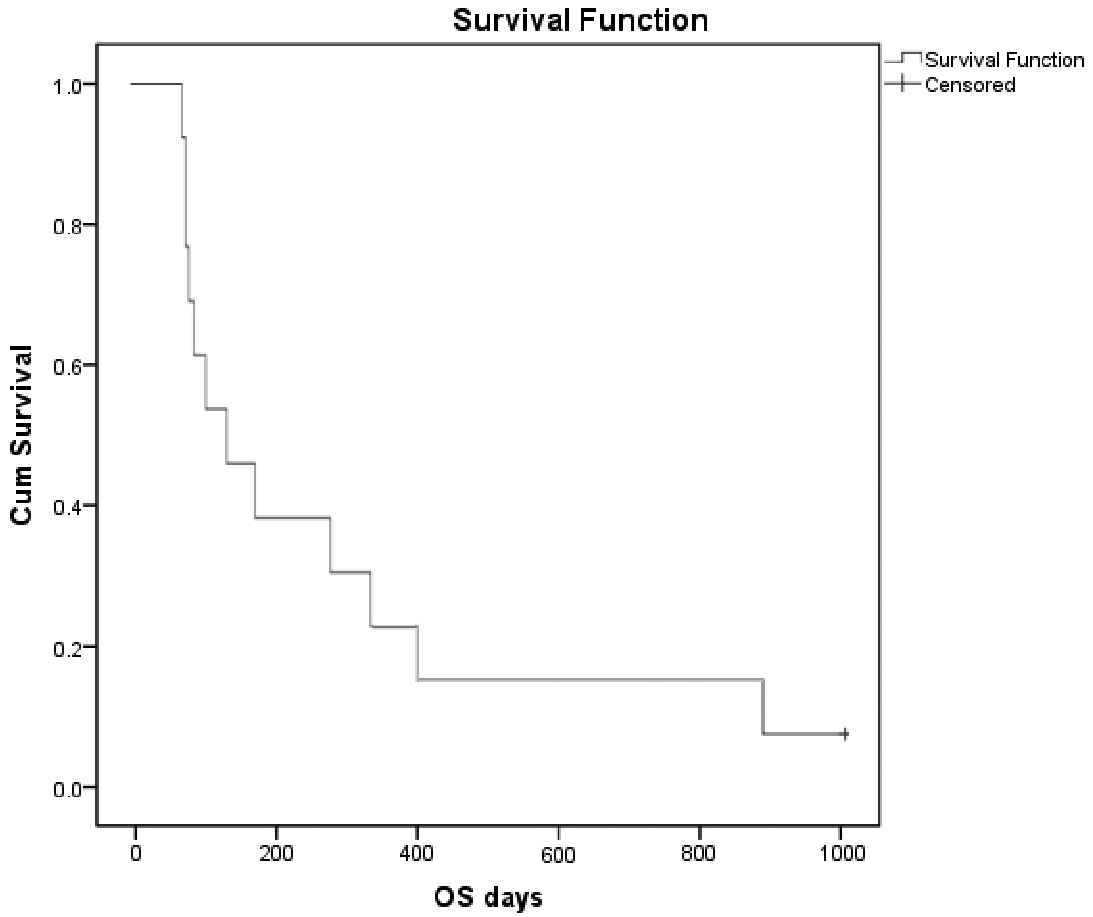

Supplementary Figure S5: **Survival plot of the 13 Ad5/3-E2F-Δ24-GMCSF treated patients. Median survival was 104 days. One patient is still alive after 1000 days follow up.**

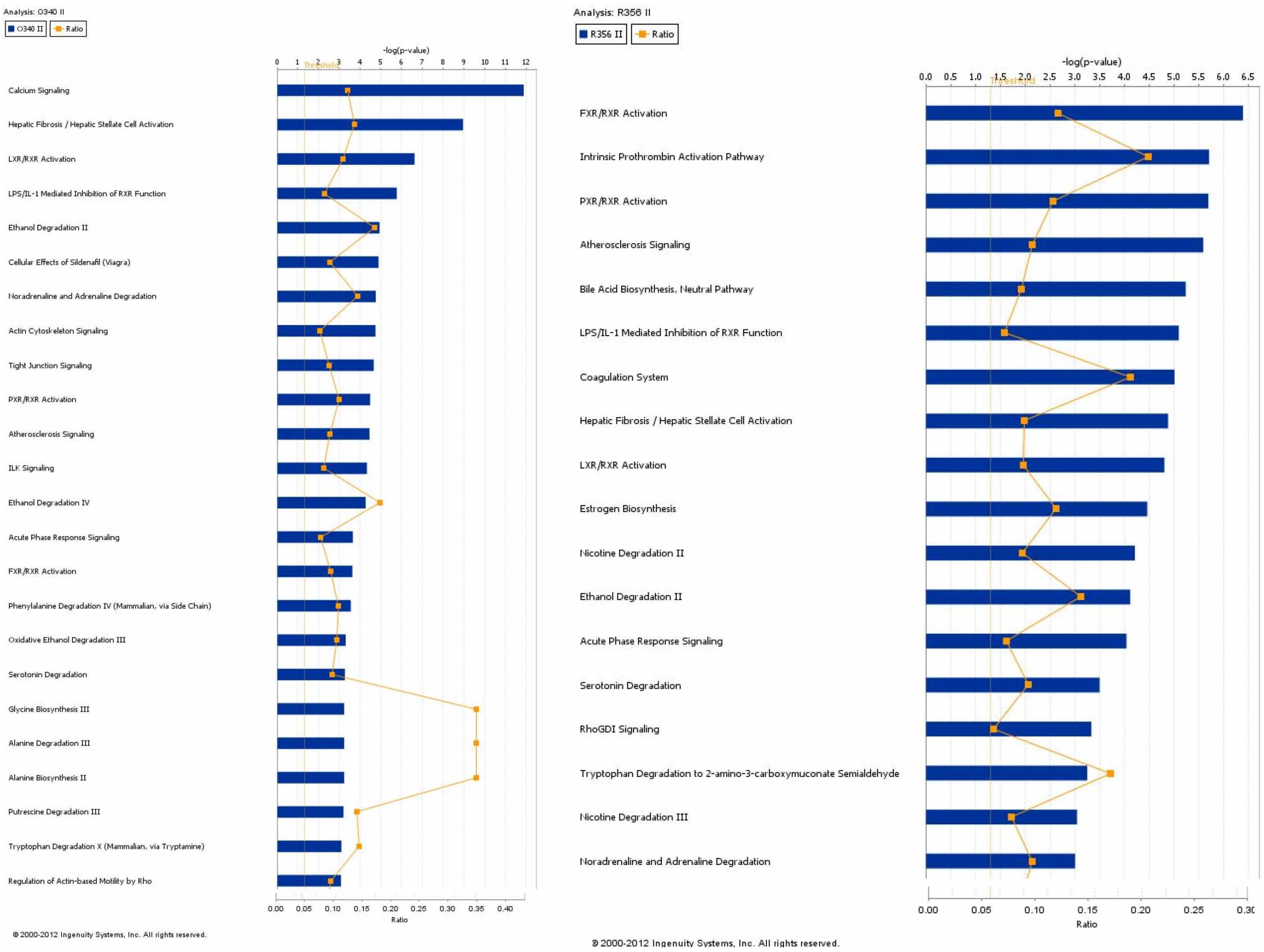

Supplementary Figure S6: RNA expression patterns were analysed by Ingenuity pathway analysis software (Ingenuity System Inc, USA) significantly altered patways are shown. Interestingly, nine of these pathways were the same with both patients, and many associate with immunological activity.

Supplementary Table S1: Prior anti-cancer therapies

Patient ID	Prior therapies
O314	surgery, docetaxel + carboplatin (x2), pegylated doxorubicin hydrochloride, paclitaxel, carboplatin, gemcitabine, topotecan
O337	surgery x 2, carboplatin + paclitaxel, carboplatin (x2), radiotherapy, carboplatin, topotecan, pegylated doxorubicin hydrochloride, tamoxifen
O340	surgery (x5), bleomycin, etoposide, cisplatin, letrozole
O351	surgery, paclitaxel + carboplatin (x3), gemcitabine + carboplatin, gemcitabine, pegylated doxorubicin hydrochloride, topotecan, etoposide, oxaliplatin, vinorelbin, tamoxifen
C312	capecitabine, capecitabine + oxaliplatin, capecitabine + oxaliplatin + bevacizumab, irinotecan + bevacizumab, bevacizumab, cetuximab + 5-fluorouracil + irinotecan (x2), oxaliplatin + 5-fluorouracil, bevacizumab
C332*	surgery x 2, capecitabine + oxaliplatin + bevacizumab, capecitabine + irinotecan + bevacizumab, capecitabine + bevacizumab
H192	gemcitabine, capecitabine, gemcitabine chemoradiation, gemcitabine + erlotinib

(*Continued*)

Patient ID	Prior therapies
H344	surgery, gemcitabine + erlotinib, gemcitabine + capesitabine, gemcitabine + oxaliplatin + capesitabine, gemcitabine + erlotinib
I347	surgery, dacarbazine + interferon, paclitaxel + carboplatin
R319	docetaxel (multiple), gemcitabine, cyclophosphamide + epirubicin + 5-fluorouracil, capecitabine, vinorelbine + epirubicin + 5-fluorouracil , doxorubicin, toremifene citrate, letrozole, fulvestrant, exemestane, medroxyprogesterone
R342	surgery x2, cyclophosphamide, epirubicin, 5-fluorouracil, radiotherapy, docetaxel + capecitabine, capecitabine, pegylated doxorubicin hydrochloride, cisplatin + gemcitabine
R356	surgery x4, radiotherapy x 2, cyclophosphamide + epirubicin + 5-fluorouracil, vinorelbine + trastuzumab, capecitabine + lapatinib, paclitaxel + bevacizumab, docetaxel + epirubicin + capecitabine + bevacizumab, paclitaxel + gemcitabine
S352	surgery x 2
S354	surgery, ifosfamide + doxorubicin (x6), radiotherapy

*Underwent hyperthermic intraperitoneal chemotherapy (HIPEC) surgery 3 weeks later and was subsequently treated with other viruses and is thus included only for immunohistochemistry analysis from the sample obtained from surgery. In long-term safety follow-up, it was discovered that the patient (who had ulcerative colitis as a baseline condition) was still alive and well three years after virus treatment. However, she had had a second primary colorectal cancer which was detected 2 years 2 months after the peritonectomy procedure. She then underwent proctocolectomy and splenectomy and pathological analysis revealed a stage II (T3N0M0) colon tumor but there was also a surprise finding of low grade B-cell lymphoma in the spleen. While both colorectal cancer and lymphoma are known sequelae of ulcerative colitis(Lenzen, Borchard et al. 1995; Jess, Rungoe et al. 2012), it cannot be outruled at this point that virus treatment affected these diagnoses in some manner.

Jess, T., C. Rungoe. "Risk of colorectal cancer in patients with ulcerative colitis: a meta-analysis of population-based cohort studies." Clin Gastroenterol Hepatol et al. 2012; 10:639–645.

BACKGROUND & AIMS: Patients with ulcerative colitis (UC) have an increased risk of developing colorectal cancer (CRC). Studies examining the magnitude of this association have yielded conflicting results. We performed a meta-analysis of population-based cohort studies to determine the risk of CRC in patients with UC. METHODS: We used MEDLINE, EMBASE, Cochrane, and CINAHL to perform a systematic literature search. We included 8 studies in the meta-analysis on the basis of strict inclusion and exclusion criteria. We calculated pooled standardized incidence ratios (SIRs) with 95% confidence intervals (CIs) for risk of CRC in patients with UC and performed meta-regression analyses of the effect of cohort size, calendar period, observation time, percentage with proctitis, and rates of colectomy on the risk of CRC. RESULTS: An average of 1.6% of patients with UC was diagnosed with CRC during 14 years of follow-up. SIRs ranged from 1.05 to 3.1, with a pooled SIR of 2.4 (95% CI, 2.1–2.7). Men with UC had a greater risk of CRC (SIR, 2.6; 95% CI, 2.2–3.0) than women (SIR, 1.9; 95% CI, 1.5–2.3). Young age was a risk factor for CRC (SIR, 8.6; 95% CI, 3.8–19.5; although this might have resulted from small numbers), as was extensive colitis (SIR, 4.8; 95% CI, 3.9–5.9). In meta-regression analyses, only cohort size was associated with risk of CRC. CONCLUSIONS: In population-based cohorts, UC increases the risk of CRC 2.4-fold. Male sex, young age at diagnosis with UC, and extensive colitis increase the risk.

Lenzen, R., F. Borchard. "Colitis ulcerosa complicated by malignant lymphoma: case report and analysis of published works." Gut et al. 1995; 36:306–310.

A 51 year old woman with a two year history of ulcerative colitis developed a wide spread gastrointestinal non-Hodgkin's lymphoma of low grade malignancy (MALT-lymphoma) involving upper and lower gastrointestinal tract, spleen, and bone marrow. After chemotherapy, clinical symptoms improved and lymphocytic infiltrates disappeared. Thirty nine cases of ulcerative colitis and 22 cases of Crohn's disease complicated by gastrointestinal lymphomas reported in published works are reviewed. In inflammatory bowel diseases any dense lymphocytic infiltrates seen in biopsy specimens obtained from ulcerative colitis or Crohn's disease should be assessed to exclude gastrointestinal lymphoma.

Supplementary Table S2: Adverse events

	Grade 1	Grade 2	Grade 3	Grade 4–5
Constitutional				
Fever	11/39	11/39	2/39	0/39
Fatigue	4/39	18/39	0/39	0/39
Chills	0/39	1/39	0/39	0/39
Rigors	3/39	1/39	0/39	0/39
Gastrointestinal				
Anorexia	2/39	4/39	0/39	0/39
Nausea	5/39	6/39	0/39	0/39
Vomiting	5/39	2/39	0/39	0/39
Heartburn	3/39	0/39	0/39	0/39
Diarrhea	1/39	2/39	0/39	0/39
Constipation	2/39	1/39	0/39	0/39
Distension	2/39	1/39	0/39	0/39
Ileus	0/39	1/39	0/39	0/39
Feel of satietas	0/39	1/39	0/39	0/39
Neurologic and Ocular				
Neuropathy	2/39	0/39	1/39	0/39
Muscle cramps	1/39	0/39	0/39	0/39
Pain				
Injection site	5/39	1/39	0/39	0/39
Abdominal	7/39	8/39	1/39	0/39
Limbs	1/39	5/39	0/39	0/39
Back	0/39	2/39	0/39	0/39
Chest wall	1/39	0/39	1/39	0/39
Headache	3/39	1/39	0/39	0/39
Flank	0/39	2/39	1/39	0/39
Others	7/39	6/39	2/39	0/39
Pulmonary/Upper respiratory				
Pneumothorax	1/39	0/39	0/39	0/39
Dyspnea	2/39	2/39	1/39	0/39
Cough	1/39	1/39	0/39	0/39
Lung infection	0/39	0/39	1/39	0/39
Hematological				
Anemia	5/39	10/39	2/39	0/39
Leukocytopenia	4/39	3/39	1/39	0/39
Thrombocytopenia	7/39	0/39	0/39	0/39

(*Continued*)

	Grade 1	Grade 2	Grade 3	Grade 4–5
Metabolic/Laboratory				
AST elevation	10/39	1/39	0/39	0/39
Hypokalemia	1/39	0/39	0/39	0/39
Hyponatremia	6/39	0/39	2/39	0/39
Creatinine elevation	0/39	0/39	1/39	0/39
INR elevation	1/39	0/39	0/39	0/39
Other				
Oedema	3/39	3/39	1/39	0/39
Urine incontinence	0/39	1/39	0/39	0/39
Hemorrhage	2/39	0/39	0/39	0/39
Flu-like symptoms	1/39	2/39	0/39	0/39
Flushing	0/39	0/39	1/39	0/39
Alopecia	1/39	0/39	0/39	0/39
Pruritus	0/39	1/39	0/39	0/39
Voice changes	1/39	0/39	0/39	0/39

Serious adverse events that were possibly treatment related and needed hospitalization were the following: O337 experienced pain and a tumor abscess 13 days after the third treatment, H344 had nausea, fatigue and acute renal failure (serum creatinine increased from 89 to 318) 14 days after the third treatment, O351 reported grade 3 fatigue 9 days after the third treatment, R342 experienced edema of the left arm 15 days after the first treatment and lung infection with dyspnea one day after the second treatment. Overall, treatments were well tolerated with all doses used.
